# Usefulness of Mehlich-3 test in the monitoring of phosphorus dispersion from Polish arable soils

**DOI:** 10.1007/s10661-018-6685-4

**Published:** 2018-04-19

**Authors:** Ewa Szara, Tomasz Sosulski, Magdalena Szymańska, Katarzyna Szyszkowska

**Affiliations:** Department of Soil Environment Sciences, Warsaw University of Life Sciences-SGGW, Nowoursynowska 159, 02-776 Warsaw, Poland

**Keywords:** Phosphorus, Degree of P soil saturation, Mehlich-3 extraction, Risk assessment

## Abstract

A considerable area of soils with low abundance of plant-available phosphorus and relatively low consumption of phosphorus fertilisers recorded in Poland over the last 20–25 years suggests that the dispersion of phosphates from arable soils in Poland can be low. The literature, however, provides reports on a considerable share of Polish agriculture in phosphorus pollution of Baltic Sea waters. The literature provides no data concerning phosphorus sorption parameters of arable soils in Poland. Due to this, the study involved the analysis of sorption properties: 1-point phosphorus sorption index (PSI) and degree of phosphorus saturation, based on molar ratio P, Al, and Fe determined by the Mehlich-3 method (DPS-1_M3_ = P / (Al + Fe) and DPS-2_M3_ = P / Al), 59 soils representing the main types of texture of soils in Poland, characterised by variable content of plant-available phosphorus by Egner-Riehm DL, organic carbon, and soil pH. The obtained results suggest that the soil texture has a lower effect on sorption properties (PSI) than the degree of acidification. Sorption parameters of soils increased with soil acidification as a result of an increase in the content of Al and Fe extracted by the Mehlich-3 extract in strongly acidified soils. An important finding of our study was evidencing that within the same class of abundance in plant-available phosphorus, the soils varied in the degree of phosphorus saturation and content of active phosphorus. This suggests the possibility of losses of phosphorus even from soils with low abundance of the component provided they are characterised by a high value of parameters DPS-1_M3_ and DPS-2_M3_.

## Introduction

Long-term supply of a high ratio of mineral and organic fertilisers in European agriculture has led to phosphorus accumulation in the soil and migration of significant amounts of phosphates to the hydrosphere (Sharpley 1995; Rozemeijer et al. 2014). This has imposed the need to take measures aimed at minimising the emission of this element from agricultural areas, through among others legal regulations on the maximum doses of phosphate fertilisers intended for the fertilisation of arable soils (Amery and Schoumans 2014; McDowell et al. 2017). According to Schick et al. (2013) and Rowe et al. (2017), Poland is responsible for the inflow of significant amounts of phosphates into the Baltic Sea. This may suggest the accumulation of significant amounts of phosphorus in our soils as a result of previous intensive fertilisation. However, views on the phosphorus status of cultivated soils in our country are varied (Igras and Pastuszak 2009; Sapek 2010). The fertiliser advisory system based on the phosphorus balance on ‘the surface of the field’ and soil P abundance status using Egner-Riehm’s DL test is currently the only tool that responds to the idea of sustainable agriculture (Jadczyszyn 2006). Hooda et al. (2001) and Börling et al. (2004a) stated that soils with similar levels of plant-available phosphorus can differ significantly in their ability to accumulate and release phosphorus, and therefore in their susceptibility to disperse it. This phenomenon, in addition to immobilisation and mineralisation, is mainly determined by the sorption capacity of soils resulting from the simultaneous processes of adsorption, desorption, precipitation, and dissolution taking place in the soil (Börling et al. 2001). The degree of soil P saturation is considered a reliable indicator of the risk of phosphorus losses from the soil. It is defined as the proportion of the phosphorus currently sorbed in the total sorption capacity of the soil with respect to that component. In the literature, there are a number of different ways of its determination (Beuchemin and Simirad 1999; Wang et al. 2015; Renneson et al. 2016). In a part of the USA and Canada, the degree of soil saturation with phosphorus is assessed based on relative amounts of P, Al, and Fe in the Mehlich-3 extract. The results of this test show a strong correlation with the amounts of phosphorus found in groundwater and surface runoff waters (Khiari et al. 2000; Maguire and Sims 2002; Sims et al. 2002; Pellerin et al. 2006).

In many countries, many different soil phosphorus tests are used successfully. According to Wuenscher et al. (2015), in Austria and Germany, the calcium-acetate-lactate (CAL) extract is used in routine soil testing. In Poland, the fertiliser diagnostics system is based on the phosphorus balance on ‘the surface of the field’ and soil P availability content using Egner-Riehm’s DL (Jadczyszyn 2006; Jordan-Meille et al. 2012). The National Agrochemical Station in Warsaw has recently commenced the procedure of implementation of the Mehlich-3 test as a fast, cheap, and universal method for determining the plant-available forms of K, P, and Mg in the soil (Kęsik et al. 2015). The same test is used in the Czech Republic, Estonia, Slovak Republic, and major parts of Canada and the USA (Jordan-Meille et al. 2012; Wuenscher et al. 2015). The universal character of the method makes it applicable for the assessment of agrochemical and environmental effects of fertilisation in research conducted around the globe (Paz-Ferreiro et al. 2012; Pizzeghello et al. 2016; Fukuda and Nakamura 2017).

The Melicha-3 soil test is not used to assess the state of saturation with phosphorus in arable soils of Poland, although the results obtained this way would allow the assessment of the share of Polish agriculture in the pollution of surface and groundwater with phosphates**.** The widespread use of the Mehlich-3 soil test in fertiliser diagnostics can also offer a new opportunity to implement the assessment of phosphorus saturation of arable soils on a national scale. The results obtained this way enable the provision of arguments in the still debatable issue of the share of Polish agriculture in the contamination of surface and ground waters with phosphates. Polish literature has not yet documented sufficient research results on such use of the described soil test (Szara and Sosulski 2012). In our previous work, we considered the performances and limitations of selected models and parameters for evaluation of phosphorus sorption in the soil and methodological aspects of their determination (Szara et al. 2011), or focused on the determination of the effect of application of mineral and organic fertilisers on the soil phosphorus sorption under long-term experimental conditions (Szara et al. 2017). The analysis of phosphorus sorption and saturation in numerous arable soils representing typical properties of Polish soils has not been conducted and presented in the literature so far. The objective of this study was the assessment of the possibilities and needs of use of the Mehlich-3 test for the monitoring of P sorption capacity and the degree of P saturation of typical arable soils in Poland, and determination of the dependencies of such parameters on textural classes, acidity, and plant-available phosphorus classes by Egner-Riehm DL.

## Material and methods

The study involved soil samples taken from the arable layer of cultivated soils in Poland. A total of 59 soil samples were analysed. The soil samples were air-dried and passed through a 2-mm sieve. Then, their granulometric composition was determined by the aerometric method. The results of these measurements allowed the soils to be classified into soil textural classes and agrochemical soil categories according to the Polish Society of Soil Science (Roczniki Gleboznawcze-Soil Science Annual 2009).

Soil pH was measured potentiometrically in 1 M KCl at a 1:2.5 ratio (PN-ISO 10390 1997). Total organic carbon (C_org_) was determined by means of a TOC analyser (TOC Analyser 5050A; Shimadzu, Japan), cation exchange capacity (CEC = *S* + *H*_h_) as sum of exchangeable bases sum (*S* = Na + K + Ca + Mg) Na, K, Ca, and Mg in 1 M CH_3_COONH_4_ by means of atomic absorption spectrometry (spectrometer, Thermoelemental, Solaar; England), and soil hydrolytic acidity (*H*_h_) in 0.5 M (CH_3_COO)_2_Ca. Plant-available phosphorus content was determined according to the Egner-Riehm DL metod (P_ER_) (PN-R-04023 1996). Active phosphorus content in 0.01 M CaCl_2_ at a 1:5 ratio (P_CaCl2_) was determined using the ascorbic acid method (Sharpley et al. 2008) (spectrophotometer; Genesys 10UV Thermo EC, USA).

In order to determine the degree of soil P saturation, the soil samples were subjected to 5-min extraction with the Mehlich-3 solution (M3): 0.2 M CH_3_COOH, 0.25 M NH_4_NO_3_, 0.015 M NH_4_F, 0.013 M HNO_3_, 0.001 M EDTA. The obtained extract was analysed for the amounts of aluminium (Al_M3_) and iron ((Fe_M3_) by AAS, and phosphorus (P_M3_) spectrophotometrically by the ascorbic acid method. The obtained results provided the bases for the calculation of the degree of soil P saturation: DSP-1_M3_ = P_M3_ / (Al_M3+_ Fe_M3_) and DPS-2_M3_ = P_M3_ / Al_M3_, where P_M3_, Al_M3_, and Fe_M3_ were expressed in millimoles per kilogram of soil (Sharpley et al. 2008). The analyses employed the AgroMat soil AG-2, 140025102 reference material.

One-point phosphorus sorption index (PSI) (Bache and Williams 1971) of the soils was measured by a single equilibration of 1 g of soil with 20 mL of a solution containing 25 mg P L^−1^ in 0.01 M CaCl_2_. The initial P concentration was reduced from 75 to 25 mg P L^−1^ because an excessively high dilution ratio, necessary to determine P content in the equilibration solution, did not allow the determination of the variation in P concentration in the test solutions. After 18 h of shaking, the soil samples were centrifuged and then filtered. The filtrate was analysed by the ascorbic acid method (Sharpley et al. 2008). The PSI index (L kg^−1^) was calculated as *X*/log C, where *X* was the amount of P (mg kg^−1^) sorbed by the soil, and C was P concentration (mg L^−1^) in the equilibration solution.

### Statistical analysis

The statistical analysis of the results involved the assessment of the influence of the soil agrochemical category, soil pH class, and available phosphorus class on the measured parameters: *S*, CEC, *H*_h_, C_org_, pH, PSI, P_ER_, P_M3_, Fe_M3_, Al_M3_, and saturation coefficients. Because of the varied number, variance, and distribution of the results, the Kruskal-Wallis nonparametric test was used in the statistical analysis. Spearman rank correlation coefficients (*r*) were also calculated. In each case, the pH of the soil was converted to H^+^ concentration in the soil solution. IBM SPSS Statistics 23 was used for the calculations.

## Results and discussion

The quality of Polish soils is relatively low on account of their origin, dominated by acid soil with low organic carbon and clay content. The analysed soils represented the most commonly found soil textural classes in Poland: sands (*n* = 11), loamy sands (*n* = 14), sandy loams (*n* = 12), light loams (*n* = 5), loams (*n* = 7), silty clay loams (n = 1), clay loams (n = 1), and silt loams (*n* = 8). The differences in the granulometric composition of the soil samples determined the values of the basic soil parameters. As a result, significant variations were found in the sum of exchangeable bases (*S*): 1.7–279.2 mmol(+) kg^−1^, hydrolytic acidity (*H*_h_): 8.1–94.9 mmol(+) kg^−1^, and cation exchange capacity of the soils tested (CEC): 35.2–292.8 mmol(+) kg^−1^ (Table 1). The values of the parameters mostly increased with increasing agrochemical category of the soil. The organic carbon content in the soil (C_org_) varied between 2.4 and 21.2 g kg^−1^ of soil, and did not depend on the soil agrochemical category. The tested soils were characterised by a broad pH range, from 3.3 to 7.2, and therefore, they represented pH classes from very acidic to neutral. The soil agrochemical category significantly (*p* = 0.002) differentiated the distribution of pH values for the soils tested (Table 1). The analysis of pH values showed that very light and light soils were characterised by a higher degree of acidification than medium and heavy soils.

**Table 1:** Soil properties depending on soil agrochemical category according to soil PSSS classification (2009)

	Clay < 0.002 (%)	Silt 0.05–0.002 (%)	Sand 1–0.05 (%)	pH	*H*_h_ (mmol(+) kg^−1^)	*S* (mmol(+) kg^−1^)	CEC (mmol(+) kg^−1^)	C_org_ (g kg^−1^)	PSI (L kg^−1^)
Very light soils, *n* = 11									
Mean				4.0	56.4	10.7	67.1	9.8	58.1
Median				4.6	57.8	6.4	71.3	10.5	58.9
Minimum	1	4	87	3.5	22.5	1.7	38.5	3.9	32.1
Maximum	6	11	94	5.6	87.7	35.6	101.0	13.5	95.4
Light soils, *n* = 14									
Mean				4.0	53.3	23.1	76.4	9.9	69.7
Median				4.3	52.7	12.8	74.6	8.7	72.8
Minimum	3	5	73	3.3	20.8	2.5	51.1	4.1	13.3
Maximum	9	20	88	6.7	94.9	81.4	106.3	21.2	145.6
Medium soils, *n* = 17									
Mean				4.6	31.8	73.4	105.1	9.3	74.1
Median				5.6	30.0	46.9	80.4	8.9	80.6
Minimum	2	13	58	3.6	11.8	10.4	34.2	2.4	20.0
Maximum	14	36	78	7.3	63.8	223.8	235.2	16.2	165.2
Heavy soils, *n* = 17									
Mean				4.8	24.6	130.6	155.2	11.7	74.3
Median				6.1	16.6	128.5	148.3	12.5	70.9
Minimum	2	30	12	3.7	8.1	10.9	59.0	4.7	25.1
Maximum	29	68	51	7.2	67.1	279.2	292.8	16.4	179.2
*p*^a^				0.002	0.001	0.001	0.002	0.241	0.744
Overall, *n* = 59									
Mean				4.3	39.4	66.3	105.7	10.2	70.3
Median				5.0	33.1	35.2	81.5	10.5	66.7
Minimum	1	4	12	3.3	8.1	1.7	35.2	2.4	13.3
Maximum	29	68	94	7.2	94.9	279.2	292.8	21.2	179.2
PSI (*r*)^a^	− 0.013	0.008	0.009	0.360**	0.324*	− 0.239	− 0.098	− 0.261*	

^a^Significance of the Kruskal-Wallis test for the soil category factor

^b^Spearman rank correlation coefficient; significant for *p < 0.05 or **p < 0.01

Studies on the sorption properties of phosphorus in arable soils in Poland are currently scarce. They indicate rather low phosphorus sorption capacity in our soils (Szara et al. 2011, 2017). The 1-point sorption index (PSI) of the tested soils ranged from 13.3 to 179.2 L kg^−1^ of soil and did not depend on their granulometric composition (Table 1). The PSI index is not a direct measure of the sorption capacity of soils with respect to phosphorus, but it correlates significantly with the sorption capacity determined based on sorption isotherms and allows comparison of sorption capacities of soils (Börling et al. 2001). As shown by Zhang et al. (2005), the effect of soil silt content on the sorption capacity for phosphorus is indirect and generally cannot be determined by simple statistical methods. Our results show, however, that the mean and median PSI of very light soils are markedly lower than those for light, medium, and heavy soils. The PSI sorption index showed a weak but significant correlation with H^+^ concentration calculated from soil pH (*r* = 0.360**). Both the mean (70.3 L kg^−1^) and median (66.7 L kg^−1^) values of PSI were highest for strongly acidic soils (Table 2). For neutral soils, the median and mean values of PSI were significantly lower: 47.2 and 48.6 L kg^−1^, respectively. Significant Spearman rank correlation was also determined for PSI values and C_org_ content in the soils (*r* = − 0.261*). The negative value of the correlation coefficient suggests that an increase in soil organic carbon content could reduce the sorption capacity of the tested soils for phosphorus. This dependence is known from the literature. The primary known reason for this phenomenon is the use of organic fertilisers. Low-molecular-weight organic compounds resulting from the decomposition of such fertilisers can exhibit a high degree of competitiveness in the occupation of sorption sites in relation to phosphates, or, by complexing Al and Fe, they limit their activity in the soil solution (Iyamuremye et al. 1996; You et al. 2007).

**Table 2:** Soil properties depending on soil pH class (PN-ISO 10390 1997)

	Fe_M3_ (mmol kg^−1^)	Al_M_ (mmol kg^−1^)	Fe_M3_ + Al_M3_ (mmol kg^−1^)	PSI (L kg^−1^)
*n* = 20	Very acid soil; pH_KCl_ < 4.5			
Mean	4.6	18.3	22.8	81.2
Median	4.2	16.8	21.7	79.3
Minimum	1.3	10.7	12.9	32.1
Maximum	9.5	30.0	35.3	179.2
*n* = 14	Acid soil; pH_KCl_ 4.5–5.5			
Mean	3.2	14.8	18.0	70.2
Median	2.6	13.1	16.1	67.9
Minimum	1.8	6.8	10.5	25.3
Maximum	8.5	22.7	25.8	145.5
*n* = 14	Medium acid soil; pH_KCl_ 5.6–6.5			
Mean	2.8	13.1	15.9	71.9
Median	2.7	12.2	14.4	70.0
Minimum	1.1	2.1	6.0	13.3
Maximum	4.2	23.0	26.8	165.2
*n* = 11	Neutral soil; pH_KCl_ 6.6–7.2			
Mean	2.4	7.4	9.8	48.6
Median	2.5	6.9	9.3	47.2
Minimum	0.2	2.5	5.5	20.0
Maximum	3.7	11.9	14.5	82.5
*p*^a^	0.017	< 0.0001	< 0.0001	0.062
*n* = 59	Overall			
Mean	3.4	14.2	17.6	70.3
Median	2.9	13.1	15.4	66.7
Minimum	0.2	2.1	5.5	13.3
Maximum	9.5	30.0	35.3	179.2
PSI (*r*)^b^	0.313**	0.495**	0.542**	
pH (*r*)^b^	0.409**	0.666**	0.692**	0.360*

^a^Significance of the Kruskal-Wallis test for the soil pH factor

^b^Spearman rank correlation coefficient; significant for *p < 0.05 or **p < 0.01

The dominant role in the determination of the phosphorus sorption capacity of mineral soils with varying degrees of acidification is played by amorphous and poorly crystallised, and hydroxyl-iron and aluminium oxides (Lookman et al. 1996). The classic method of determining their amounts in the soil is extraction with ammonium oxalate. The combined Fe and Al content determined by this method shows a strong correlation with sorption indices based on sorption isotherms (Zhang et al. 2005; Szara et al. 2011).The degree of soil P saturation determined based on the amounts of Fe, Al, and P in ammonium oxalate solution acts as a reference indicator of the susceptibility of non-alkaline soils to phosphorus dispersion from soils to surface and ground waters (Pautler and Sims 2000; Börling et al. 2004a). The Mehlich-3 solution exhibits lower specificity for non-crystallised forms of iron than aluminium. According to Maguire and Sims (2002), the amount of Fe_M3_ constitutes only about 30% of forms extracted with ammonium oxalate. According to Monterroso et al. (1999), Fe_M3_ exhibits a greater correlation with available, exchangeable, and soluble forms of iron than with poorly crystallised and amorphous forms of this element. Fe_M3_ content in the analysed soils ranged from 0.2 to 9.5 mmol kg^−1^ of soil and, like the Al_M3_ content, was positively correlated with H^+^ concentration in the soil solution calculated from pH (Table 2). The amount of both Al_M3_ and Fe_M3_ was significantly correlated with the PSI sorption index (Table 2). The significant contribution of Al_M3_ in the determination of soil sorption capacity for phosphorus is found within a wide range of their pH values: from acidic to neutral (Kleinman and Sharpley 2002). In the case of Fe_M3_, such a correlation is less frequent (Zhang et al. 2005). Obtaining a significant correlation between PSI and Fe_M3_ content in the analysed soils may have been determined by the predominant share of very acidic and acidic soils. Strong acidification of soils is conducive to the appearance of mobile forms of iron that pass most effectively into the Mehlich-3 solution. This in turn could have resulted in obtaining an average Fe_M3_ contribution of about 20% in ∑(Fe_M3_, Al_M3_). This result is similar to that obtained by Sims et al. (2002) for the Mid-Atlantic soils in the USA (19%), but greater than that obtained for the acidic soils of Canada (12%) (Khiari et al. 2000).

The tested soils were characterised by highly varied amounts of plant-available phosphorus, estimated by the standard (in Poland) Egner-Riehm’s method (P_ER_), from 4.6 to 238.1 mg P kg^−1^ of soil. They represented all classes of P abundance (Table 3). The amount of plant-available phosphorus extracted with the Mehlich-3 solution (P_M3_) showed even greater variation, ranging from 2.6 to 518.2 mg P kg^−1^ of soil (Table 3). The amounts of phosphorus obtained with the Mehlich-3 solution are generally higher than those in the double calcium lactate solution (Szara and Sosulski 2012; Eriksson et al. 2013).

**Table 3:** Soil properties depending on the Polish class of plant-available phosphorus by Egner-Riehm DL (PN-R-04023 1996)

	P_ER_ (mg kg^−1^)	P_M3_ (mg kg^−1^)	DPS-1_M3_	DPS-2_M3_	P_CaCl2_ (mg kg^−1^)
*n* = 9; very low class of available phosphorus < 22 mg P_ER_ kg^−1^					
Mean	13.1	19.5	0.081	0.067	1.49
Median	12.3	6.0	0.099	0.064	1.24
Minimum	4.6	2.6	0.007	0.010	0.36
Maximum	21.3	58.3	0.131	0.180	3.35
*n* = 10; low class of available phosphorus < 22–45) mg P_ER_ kg^−1^					
Mean	31.9	36.8	0.111	0.081	1.28
Median	31.0	38.0	0.114	0.073	0.88
Minimum	26.2	4.9	0.057	0.010	0.65
Maximum	38.5	66.2	0.179	0.170	2.85
*n* = 8; medium class of available phosphorus < 45–66) mg P_ER_ kg^−1^					
Mean	52.8	74.9	0.179	0.154	1.92
Median	52.0	61.9	0.172	0.111	1.73
Minimum	45.5	6.4	0.084	0.010	1.02
Maximum	60.0	204.9	0.318	0.370	3.13
*n* = 9; high class of available phosphorus < 66–88) mg P_ER_ kg^−1^					
Mean	77.0	96.1	0.243	0.270	3.54
Median	77.9	116.6	0.218	0.241	3.43
Minimum	68.6	7.6	0.076	0.100	1.64
Maximum	84.9	202.5	0.423	0.500	5.54
*n* = 23; very high class of available phosphorus > 88 mg P_ER_ kg^−1^					
Mean	147.5	173.1	0.455	0.642	5.78
Median	141.8	141.0	0.417	0.502	5.53
Minimum	93.3	9.7	0.052	0.010	1.02
Maximum	238.1	518.2	1.279	3.130	11.34
*p*^a^	< 0.001	< 0.0001	< 0.0001	< 0.0001	< 0.0001
*n* = 59; overall					
Mean	83.8	66.5	0.268	0.336	5.78
Median	72.2	102.2	0.182	0.187	5.53
Minimum	4.6	2.6	0.007	0.010	0.36
Maximum	238.1	518.2	1.179	3.130	11.32
P_CaCl2_ (*r*)^b^	0.770**	0.573**	0.845**	0.628**	

^a^Significance of Kruskal-Wallis test for P abundance factor

^b^Spearman rank correlation coefficient; significant for *p < 0.05 or **p < 0.01

The degree of soil P saturation (DPS-1_M3_) based on the amounts of Fe_M3_, Al_M3_, and P_M3_ in the analysed soils ranged from 0.007 to 1.179 (Table 3). Because the phosphorus content measured by the Egner-Riehm (P_ER_) method was strongly correlated (*r* = 0.734**) with the P content measured by the Mehlich-3 (P_M3_) method (Table 2), the distribution of DSP-1_M3_ values for soils belonging to different classes of available phosphorus was significantly different (Table 3). Both the mean and median DSP-1_M3_ increased with the increase in abundance of plant-available phosphorus in soils. However, within the same classes of P content, the soils differed significantly in the saturation index and therefore, in the potential susceptibility to phosphorus dispersion. This confirms the view that the usefulness of agrochemical tests used for fertiliser diagnostics is rather limited or even insufficient for the assessment of the environmental effects of phosphorus fertilisation (Pautler and Sims 2000; Hooda et al. 2001).

Irrespective of the method of determination of the degree of soil P saturation, it is important for this amount to be significantly correlated with the amount of phosphorus leached into the groundwater and/or surface waters. An indirect indicator of the amount of phosphorus lost from the soil into waters can be the amount of active phosphorus in the soil (Börling et al. 2004b). The active phosphorus content (P_CaCl2_) in the tested soils ranged from 0.36 to 11.32 mg P kg^−1^ and showed a significant strong correlation with the amounts of phosphorus forms available to plants measured by the Egner-Riehm DL (P_ER_) and Mehlich-3 (P_M3_) methods. The P_CaCl2_ content also showed a very strong Spearman rank correlation (*r* = 0.845**) with the degree of soil P saturation (DPS-1_M3_). Due to the small amounts of iron estimated in the Mehlich-3 solution in the soils of Canada and the USA, it has also been proposed to omit content of Fe_M3_ when determining the saturation coefficient (DPS-2_M3_). DSP-1_M3_ and DSP-2_M3_ generally show similar effectiveness in the assessment of the risk of phosphorus loss from those soils (Sims et al. 2002). In our tested soils, however, DSP-2_M3_ showed a significantly lower correlation with active phosphorus (*r* = 0.628**) than DSP-1_M3_. Therefore, the specificity of our soils and especially the considerable extent of their acidification presumably do not permit simplification of the methodology of determination of the degree of soil P saturation.

Modelling and field experiments conducted in the USA and Canada have resulted in the determination of the critical level of soil P saturation. Exceeding this level causes a dramatic increase in the amount of phosphorus lost from the soil. The critical DSP-1_M3_ value established for those countries is in the range 0.10–0.15 (Sims et al. 2002). If the upper limit of this range is taken as a reference value for the assessment of the environmental effects of phosphorus management in Poland, almost all the analysed soils with very high and high P abundance (30 out of 32) were characterised by a DSP-1_M3_ value exceeding the critical value, not infrequently in a considerable way (more than threefold). In five out of eight soils with a medium phosphorus content, DSP-1_M3_ assumed values in a range of 0.16–0.29. These above-the-limit values of DSP-1_M3_ in the tested soils raise doubts whether the principle of aiming for and maintaining medium soil fertility with respect to phosphorus (P_ER_) adopted in our fertiliser advisory system is an optimal solution reconciling production and environmental requirements. Our results suggest that the existing fertiliser recommendations have promoted phosphorus dispersion into the environment. This dispersion may have been particularly intense on farms where soil fertility was built up by intensive organic fertilisation (Leinweber et al. 1997; Schneider et al. 2016). On the other hand, the obtained results indicate the need to develop original critical DSP-1_M3_ values for Poland correlated with the amount of phosphorus flowing out of arable soils.

## Conclusions

In arable soils with varying physicochemical properties typical of the conditions in Poland, the amount of aluminium and iron found in the Mehlich-3 solution was significantly positively correlated with the sorption properties of the soils expressed by the one point sorption index (PSI). Their amount increased with soil acidification.

The amount of phosphorus extracted with the Mehlich-3 solution for the majority of soils was higher than the amount extracted with double calcium lactate, but the amounts of phosphorus extracted with the two solutions were significantly correlated with each other.

Both of the degrees of soil P saturation (DPS-1_M3_ and DSP-2 _M3_) based on the Mehlich-3 strongly correlated with the active phosphorus content (P_CaCl2_), but DPS-1_M3_ based on the amounts of Fe_M3_, Al_M3_, and P_M3_ showed more promise for identifying soils susceptible to phosphorus dispersion in Polish soil conditions.

Soils characterised by similar abundance of available phosphorus (P_ER_) differed in the degree of soil P saturation (DSP-1_M3_) depending on the combined amount of Fe and Al in the Mehlich-3 solution. The highest values of DPS-1_M3_ were obtained for soils of high and very high P abundance. However, significant fluctuations in this parameter within the remaining P abundance classes indicate the need to expand the monitoring of the soil phosphorus status by the phosphorus saturation index and its inclusion in the fertiliser advisory system. It is necessary, however, to develop original critical DSP-1_M3_ values for Poland, particularly taking into account the degree of acidification.
